# Hybridization in bottlenose dolphins—A case study of *Tursiops aduncus* × *T*. *truncatus* hybrids and successful backcross hybridization events

**DOI:** 10.1371/journal.pone.0201722

**Published:** 2018-09-12

**Authors:** T. Gridley, S. H. Elwen, G. Harris, D. M. Moore, A. R. Hoelzel, F. Lampen

**Affiliations:** 1 Centre for Statistics in Ecology, Environment and Conservation, Department of Statistical Sciences, University of Cape Town, C/o Sea Search Research and Conservation NPC, Muizenberg Cape Town, South Africa; 2 Mammal Research Institute, Department of Zoology and Entomology, University of Pretoria, C/o Sea Search Research and Conservation NPC, Muizenberg Cape Town, South Africa; 3 The South African Association for Marine Biological Research, uShaka Sea World, Point, Durban, South Africa; 4 Department of Biosciences, Durham University, Durham, United Kingdom; University of Missouri Columbia, UNITED STATES

## Abstract

The bottlenose dolphin, genus *Tursiops* is one of the best studied of all the Cetacea with a minimum of two species widely recognised. Common bottlenose dolphins (*T*. *truncatus*), are the cetacean species most frequently held in captivity and are known to hybridize with species from at least 6 different genera. In this study, we document several intra-generic hybridization events between *T*. *truncatus* and *T*. *aduncus* held in captivity. We demonstrate that the F_1_ hybrids are fertile and can backcross producing apparently healthy offspring, thereby showing introgressive inter-specific hybridization within the genus. We document that female F_1_ hybrids can reach sexual maturity at 4 yr and 3 mo of age, and can become pregnant and give birth before being fully weaned. The information presented has implications for understanding hybrid reticulation among cetacean species and practical implications for captive facilities housing either *Tursiops* species or hybrids thereof.

## Introduction

It is becoming increasingly clear that reticulation among species lineages is common [1], and can even support the establishment of new species radiations [2]. In her 2009 review of hybridization events in marine mammals, Bérubé [3], summarises that 53 putative hybridization events have been reported within Cetacea, of which 28 hybrids have been identified within captive facilities. The evolutionary significance of hybridisation among cetacean species is not yet clear [4], however a better understanding of this process can be facilitated through investigations of hybridisation events in captivity.

The bottlenose dolphin (*Tursiops* spp.) is one of the best studied of all the cetaceans. However, there remains continued debate surrounding the number of *Tursiops* species recognised and the phylogenetic relationships between populations from which we have genetic information. In the past as many as 20 different *Tursiops* species were identified ([5] cited in [6]). In 1990, Ross and Cockcroft [6] re-assessed the genus *Tursiops* and recognised only *T*. *truncatus*, with high degrees of morphological variation linked to clines in sea surface temperate. More recently, genetic techniques in concert with morphological and osteological data, have helped to document variation in the genus at the species and population level (e.g. [7–10]). A minimum of two bottlenose dolphin species; the common bottlenose dolphin *T*. *truncatus* and Indo-Pacific bottlenose dolphin *T*. *aduncus*, are now widely accepted [11]. A third species, the Burrunan dolphin, *T*. *australis* has recently been proposed [12] and a subspecies *T*. *truncatus ponticus* is recognised from the Black Sea [13].

Hybridisation in *Tursiops* has been investigated in areas where the species ranges overlap. An early study in Taiwan based on mtDNA sequences found no evidence for introgression [8] between *T*. *aduncus* and *T*. *truncatus*, while a later study in that region using bi-parentally inherited nuclear DNA markers (20 microsatellite DNA loci) also found no evidence for admixture between the two species [14]. Off Australia, mtDNA lineages were distinct [12, 15] and there was no evidence for admixture between *T*. *truncatus* and *T*. *aduncus* lineages even when sympatric in coastal waters [16]. Although the Austral-Asian lineage of *T*. *aduncus* shows reciprocal monophyly with the South African *T*. *aduncus* lineage, they are both in the same lineage separate from *T*. *truncatus* based on a mitogenome phylogeny [9]. Estimated divergence time between *T*. *truncatus* and *T*. *aduncus* lineages was 790Ka, while the divergence between the two *T*. *aduncus* lineages was 327Ka. The divergence between these two species is relatively old within the wider delphinid radiation and while various studies have suggested polyphyly with this genus [9, 17–20], this is likely not fully resolved.

All captive hybrids are within the odontocete suborder [3]. Common bottlenose dolphins (*T*. *truncatus*) are the most frequent cetacean to be housed in captive facilities and have hybridized with species from 6 genera, including the rough toothed dolphin (*Steno bredanensis*), Guiana dolphin (*Sotalia guianensis*), Risso’s dolphin (*Grampus griseus*) and false killer whale (*Pseudorca crassidens*) [3, 21–23]. Such events may reflect naturally occurring hybridization in areas where species distributions overlap, and there is strong evidence across a range of odontocete and mystecete cetaceans for such hybridization events in the wild [17, 24–28]. However, documentation of intra-generic hybridization events in captive or free-ranging *Tursiops* are rare, possibly due to prior confusion over the taxonomic status of this genus, difficulties in identifying hybrids in the wild using morphological features, or lack of overlap in species ranges limiting opportunities for mating. Alternatively, mechanisms of reproductive isolation may be in place which actively reduce the occurrence of hybridization events within *Tursiops*.

Studies of free-ranging cetaceans have found compelling evidence that F_1_ female hybrids can be fertile and can both backcross (e.g. common minke whale x Antarctic minke whale [29], blue whale x fin whale [30]) and interbreed (e.g. Clymene dolphin [4]), which has important implications for introgressive gene flow and species evolution [1]. However, assessing the viability of F_1_ hybrids has largely been based on molecular work [31], inferred from pregnant F_1_ hybrids [29, 30] or been based on observations of F_1_ hybrids with neonatal calves [25]. Miralles *et al*., [31] identified the first hybridization event in pilot whales, between *Globicephala melas* × G. *macrorhynchus*, and provide evidence for intra-generic introgression through molecular identification of adult hybrids [32]. Interbreeding of hybrids may be responsible for the reticulate evolution of new species such as the Clymene dolphin (*Stenella clymene*) which displays a mitochondrial genome closely related to *S*. *coeruleoalba* and a nuclear genome closely related to *S*. *longirostris* [4]. Studies in captivity where animals can be closely observed provide a good opportunity to document the reproductive potential of hybrids. However, there is only one published account of a backcross being fertile. Here a *T*. *truncatus* × *Delphinus capensis* hybrid back-crossed with *T*. *truncatus* and the resulting calf died shortly after birth [33].

Before the taxonomic definitions of the *Tursiops* genus were clarified, hybridization between *T*. *t*. *gilli* (now regarded as *T*. *truncatus*) and *T*. *t*. *aduncus* (now *T*. *aduncus*) was documented [21]. The F_1_ offspring survived 5+ years in good health in Okinawa Expo Memorial Park Aquarium, Japan. More recently, Martien *et al*., [34] found molecular evidence for a *T*. *aduncus* × *T*. *truncatus* hybridization event from samples of wild animals collected near Hawaii, with STRUCTURE [35–37] analysis suggesting the sampled animal had *T*. *aduncus* ancestors at least two generations past. However, as this study was based on molecular sampling from wild animals, no mating history was available to confirm the hybrid status of the sampled individual.

Our study documents several hybridization events between *T*. *truncatus* and *T*. *aduncus* held in a single captive facility in Durban, South Africa. Best [38] provides a short description of the captive colony of *T*. *truncatus*, *T*. *aduncus* and hybrids of the two species housed in this facility. The F_1_ hybrids can be identified by their external morphological characteristics [38], however the differences are subtle. Data from this captive setting are used to unambiguously demonstrate the ability for F_1_ hybrids to produce healthy backcross hybrid offspring that live into adulthood. The results have implications for understanding the evolution of cetacean species as well as practical implications for captive facilities housing either species or hybrids.

## Methods

This study focuses on a captive colony of *T*. *truncatus*, *T*. *aduncus* and *T*. *aduncus* × *T*. *truncatus* hybrids held at uShaka Sea World (Durban, South Africa). The colony was established in 1976 within the Durban Sea World dolphinarium (a division of the South African Association for Marine Biological Research, SAAMBR). It moved to new facilities in 2004 under the name uShaka Sea World. For simplicity, we will use the current name (uShaka Sea World) to refer to the dolphinarium throughout time. It is currently the only captive facility housing dolphins in South Africa. The enclosure, some 7200 m^3^, encompasses an indoor and external holding facility and a large 3800m^3^ presentation pool. Although the seven pools in the holding facility can be separated by physical barriers, they allow visual and acoustic contact between groups. Configuration of the social groups has changed over time, and during the principle time of data collection in November 2016 the dolphins were held in three social groups, with most adult males and females held separately in two same-sex groups, and a mature *T*. *truncatus* and *T*. *aduncus* (*Tt*_1_ and *Ta*_1_) held together.

We here provide details on the breeding history, morphological characteristics (length, weight, ventral colouration pattern) and health status of this captive colony, detailing the existence of viable F_1_ *Tursiops* hybrids and a healthy backcross adult offspring. This study utilises historical medical and husbandry data collected through routine veterinary procedures and training records for the dolphins collated in November 2016. Photographs were taken in 2014 and November 2016. Updated length-weight data are summarised from March 2018, with length-weight data from the *T*. *aduncus* parent population included for comparison. No comparable length-weight data are available for the parent *T*. *truncatus* population.

Species assignment of the *T*. *aduncus* dam (*Ta*_1_) and *T*. *truncatus* sire (*Tt*_1_) of the first generation hybrids residing in uShaka Sea World was confirmed by phylogenetic analysis. DNA was extracted from blood samples preserved in 20% DMSO saturated with NaCl using a standard phenol chloroform method (after [39]). A 932bp fragment of the mtDNA control region was amplified using the forward 5' TTC TAC ATA AAC TAT TCC 3' primer and the reverse 5' ATT TTC AGT GTC TTG CTT T 3'. PCR reactions were carried out in 25μl containing 10mM Tris-HCL (pH 8.3), 50 mM KCL, 1.5 mM MgCL2, 0.2 mM dNTP, 2 mM of each primer, 10-15ng template DNA, and 0.625 U DNA Taq Polymerase (New England Biolabs, USA). The PCR cycle was 2 min at 95°C followed by 35 cycles of 40s at 95°C, 40s at 44°C, 45s at 72°C and a final extension for 10 min at 72°C. PCR products were then cleaned using the PureLink PCR Micro Kit (Invitrogen, USA). Sequencing was on an ABI 3730 and resulting sequences were analysed using Chromas 2.6.5 (https://technelysium.com.au/wp/chromas/). A neighbour joining tree was constructed using MEGA 5.2 with the Tamura-Nei evolution model (suitable given the rate variation observed across the control region) and 1000 bootstrap replications. Reference sequences were from Genbank including *T*. *truncatus* samples from the North Atlantic [40] and *T*. *aduncus* samples from South Africa and the tree was constructed using 488bp overlapping sequence from the control region Hypervariable Region 1. The outgroup chosen was *Stenella attenuata* (from [41]).

### Ethics statement

Dolphins are kept under human care under a South African Department of Environmental Affairs permit (DEA permit number withheld for confidentiality purposes). Blood samples for genetic analysis were collected during routine veterinary supervised preventative health screening procedures, performed in compliance with accredited best international welfare standards and conventions. They were collected in a voluntary manner during routine husbandry training. Other data are purely descriptive and therefore no ethics clearance was necessary. All data generated or analysed during this study are included in this published article, or are available on Genbank.

## Results

**Fig 1:**
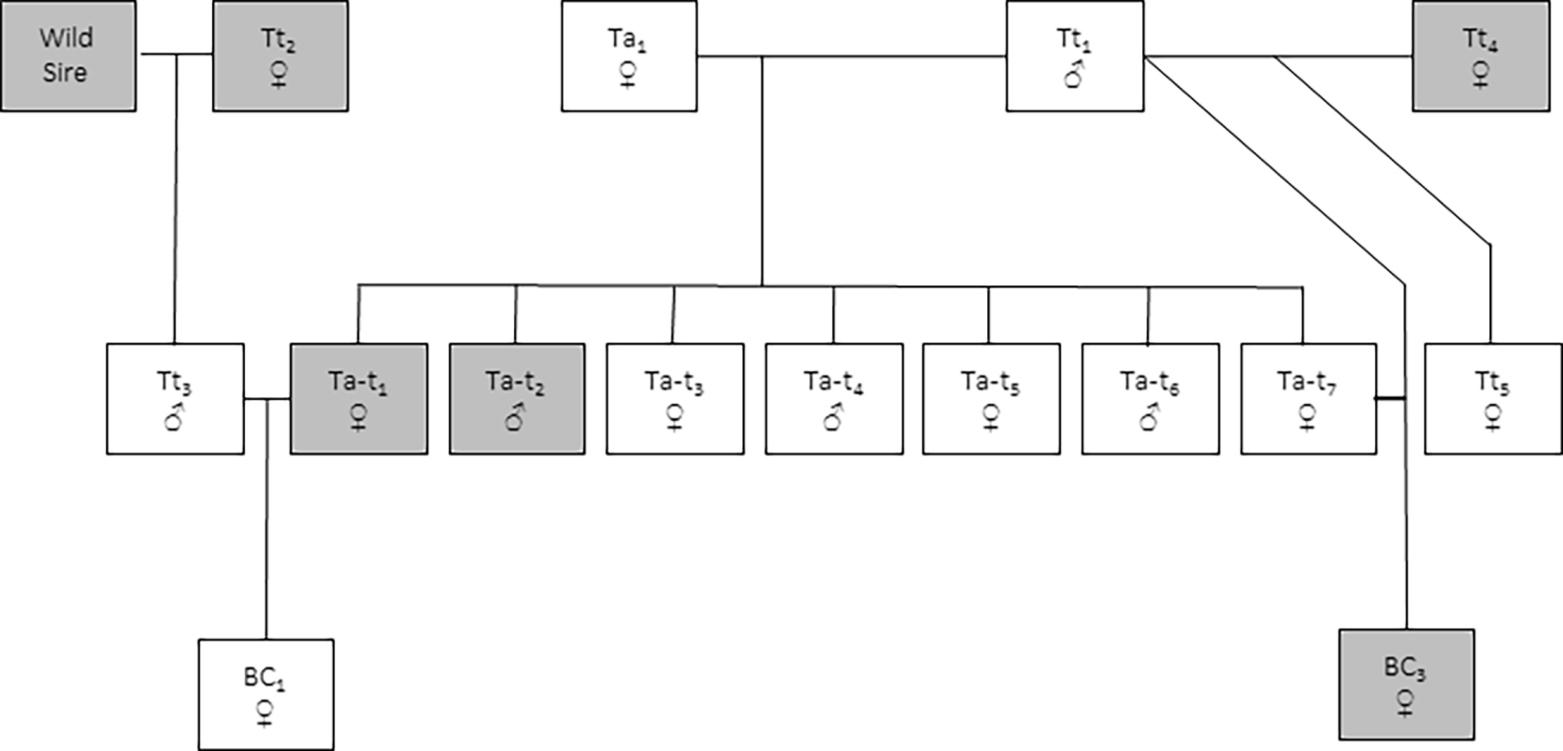
Family tree of the *Tursiops* held in the uShaka Sea World, Durban South Africa.

**Fig 2:**
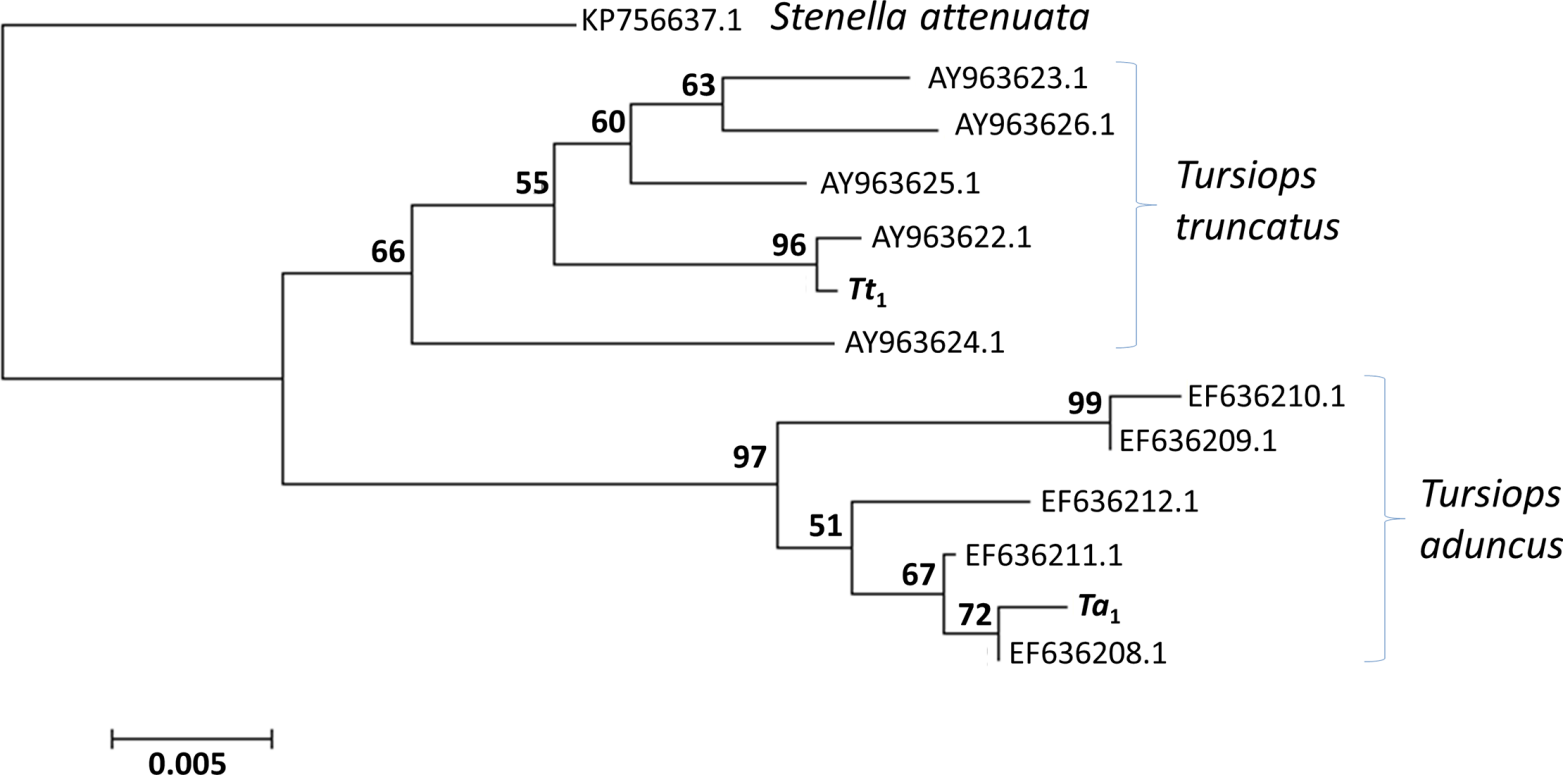
Neighbour-joining phylogeny illustrating the relationships between *Ta*_1_ and *Tt*_1_ to *T*. *aduncus* and *T*. *truncatus* specimens (NCBI accession numbers given at terminal nodes). Bootstrap values are shown based on 1000 replications.

The captive colony of *Tursiops* held at uShaka Sea World Durban includes wild stock of *T*. *truncatus* and *T*. *aduncus* captured in the southern African sub-region in the 1970s and early 1980's and their offspring born at the facility since this time (see Fig 1 and Table 1 for details). Captures of *T*. *truncatus* took place in 1976 and 1983 in Walvis Bay, Namibia (22°57’S, 14°30’E) of which *Tt*_1_ (male) is the only surviving animal. A further two pure bred *T*. *truncatus* are held: *Tt*_3_ (male) born in captivity of a pregnant wild caught dam (*Tt*_2,_ now deceased) and a wild sire, and *Tt*_5_ (female) the offspring of *Tt*_1_ and the female *Tt*_4_ (now deceased). The only pure bred *T*. *aduncus* (*Ta*_1,_ female) was captured from the waters of Umhlanga (South Africa) in 1979. Species confirmation of *Ta*_1_ and *Tt*_1_ was confirmed by lineage assignment in the mtDNA control region phylogeny (Fig 2).

**Table 1:** Background information on each bottlenose dolphin held at the uShaka Sea World.

Code	Species	Sex	Date of Capture	Date of Birth	Current status (age on 1st November 2016 or age at death)
*Ta*_1_	*Ta*	F	26/06/1979	≤ 26/06/1974*	Alive (42y, 4m)
*Tt*_1_	*Tt*	M	08/12/1976	≤ 08/12/1971*	Alive (44y, 10m)
*Tt*_2_	*Tt*	F	20/10/1983	15/06/1973*	Deceased (12y, 7m,)
*Tt*_3_	*Tt*	M	Captive born	22/01/1984	Alive (32y, 9m)
*Tt*_4_	*Tt*	F	20/10/1983	20/10/1978*	Deceased (17y, 11m)
*Tt*_5_	*Tt*	F	Captive born	12/05/1995	Alive (21y, 5m)
*Ta-t*_1_	F1 *Ta*×*Tt*	F	Captive born	23/04/1986	Deceased (9y, 1m)
*Ta-t*_2_	F1 *Ta*×*Tt*	M	Captive born	28/07/1990	Deceased (24y, 9m)
*Ta-t*_3_	F1 *Ta*×*Tt*	F	Captive born	23/05/1993	Alive (23y, 5m)
*Ta-t*_4_	F1 *Ta*×*Tt*	M	Captive born	07/09/1995	Alive (21y, 1m)
*Ta-t*_5_	F1 *Ta*×*Tt*	F	Captive born	09/12/1998	Alive (17y, 10m)
*Ta-t*_6_	F1 *Ta*×*Tt*	M	Captive born	22/05/2004	Alive (12y, 5m)
*Ta-t*_7_	F1 *Ta*×*Tt*	F	Captive born	25/11/2008	Alive (7y, 11m)
BC_1_	*Ta-t* × *Tt*	F	Captive born	17/07/1993	Alive (23y, 3m)
BC_2_	*Ta-t* × *Tt*	M	Unborn	-	Deceased (>8 m *in utero*)
BC_3_	*Ta-t* × *Tt*	F	Captive born	09/02/2014	Deceased (9d)

* Estimated from age at capture.

Periodically, since the inception of the dolphin programme, uShaka Sea World has allowed controlled breeding events to occur in the facility. In total, seven F_1_ hybrids and two backcross progeny have been born at the Sea World facilities. Of these, all the F_1_ hybrids and one calf from a backcross (paternal *T*. *truncatus*) have survived to adulthood. All F_1_ *T*. *aduncus × T*. *truncatus* hybrids held at the facility are the offspring of *Ta*_1_ and *Tt*_1_. Five out of the seven F_1_ hybrids were sired before 2000, when *T*. *truncatus* and *T*. *aduncus* were considered to be the same taxonomic species [6]. *Tt*_1_ and *Ta*_1_ are strongly bonded (as demonstrated by consistent affiliative behaviour, authors and trainers observations) and throughout time have been held together with their dependent offspring.

**Fig 3:**
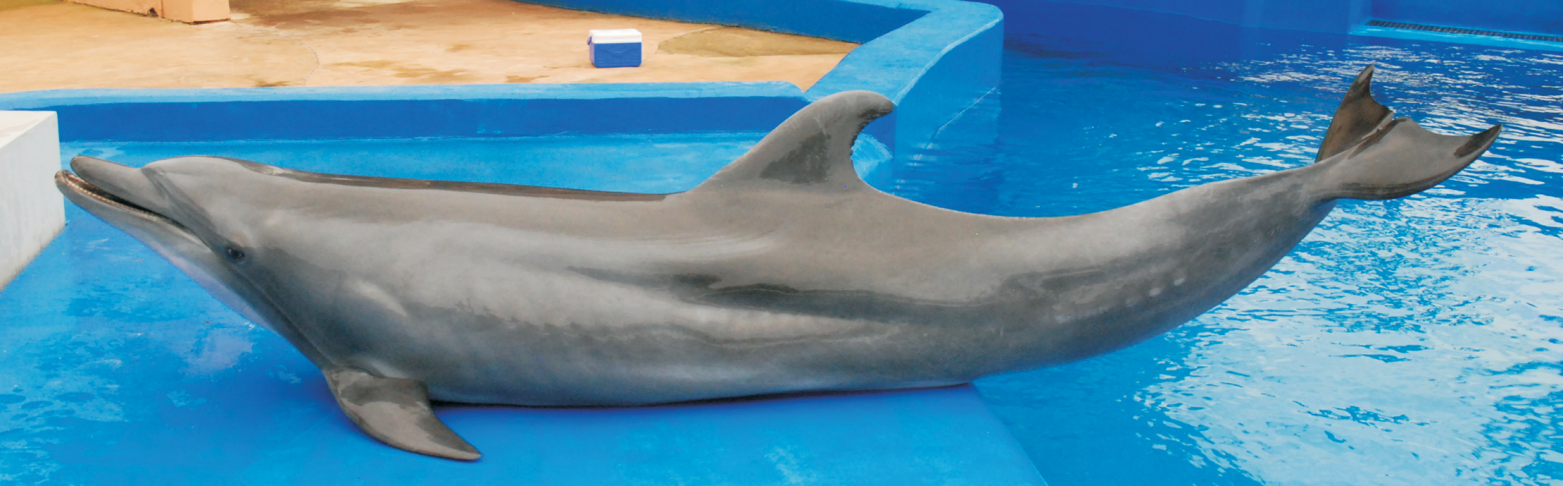
Image of BC_1_—an apparently healthy backcross hybrid at age 23 yrs.

Two backcross progeny have been born at uShaka Sea World, with a third pregnancy documented. The first backcross hybrid offspring; BC_1_, is a female and was born on the 17^th^ of July, 1993 to *Ta-t*_1_ (dam now deceased) with *Tt*_3_ the sire. The dam was an estimated 6 years and 3 months at the time of conception, based on back calculations from the date of birth (DOB) of BC_1_, using a gestation length of 12 months [42]. The BC_1_ adult is currently housed at uShaka Sea World, attaining an age of 23 years in 2016 and currently (2018) weighing 240.5 kg (Fig 3). Regular veterinary monitoring demonstrates that BC_1_ is a healthy individual and ultrasound examinations indicate normal ovulation activity in this female.

A second pregnancy was documented in *Ta-t*_1_ (foetus hereby referred to as BC_2_), representing another backcross event with *Tt*_3_. Of note is that *Ta-t*_1_ was lactating at the time of conception, with BC_1_ who was two years old during this time period observed suckling. However, *Ta-t*_1_ died on the 30th of May 1995 (at age 9 years) whilst pregnant with the unborn male calf *in utero*. She was estimated to be in the third trimester of pregnancy at the time of her death. The cause of death for *Ta-t*_1_ and associated unborn calf (BC_2_) was a peracute infection, possibly caused by the bacterium *Clostridium chauvoei*, resulting in toxaemia. The autopsy report states that the foetus and amniotic fluid appeared normal.

The second backcross (BC_3_) offspring born at uShaka Sea World was born to *Ta-t*_7_ on the 9^th^ of February 2014. *Ta-t*_7_ is estimated to have been 4 years and 3 months old at the time of conception (again back calculated from the DOB of BC_3_) and demonstrated no obvious behaviour or physical signs to demonstrate reproductive receptivity. At the time of conception she was physically small, weighing around 222 kg (weight as of February 2013) and had no clear pattern of ventral speckling—a sign of physical maturation in some *Tursiops* species [6, 43]. Although fed on a diet of fish and squid from April 2009 onwards, she continued to suckle milk from her mother. As such, she was housed in a social unit consisting of *Ta*_1_ and *Tt*_1_, her biological mother and father. Copulation was not observed but as they were housed together, it is most likely that *Tt*_1_ sired BC_3_, as all other males were held together in adjacent pools, with no free intermixing between groups taking place. Pregnancy was confirmed in *Ta-t*_7_ during a routine ultra sound examination on the 14th June 2013 and she was carefully monitored thereafter. Body length measured around this time in 2014 was estimated at 2.65 m i.e. longer than her thoroughbred mother (*Ta*_1_) but shorter than the adult hybrids. *Ta-t*_7_ continued to grow by an est. 26 cm in the following years, attaining an adult length of 2.91 m in 2018 (Fig 3).

No abnormal behaviour or physical symptoms were demonstrated during *Ta-t*_7_'s pregnancy. When born, BC_3_ was closely observed and appeared healthy, although for managerial reasons no individual medical examinations were conducted with BC_3_. In the days following birth, BC_3_ suckled from both her mother (*Ta-t*_7_) and maternal grandmother (*Ta*_1_). BC_3_ died on the 18th of February at 9 days old. Post mortem examinations revealed BC_3_ suffered nutritional complications, most likely resulting from a lack of sufficient colostrum intake in the days following birth and an associated undetermined infection.

**Fig 4:**
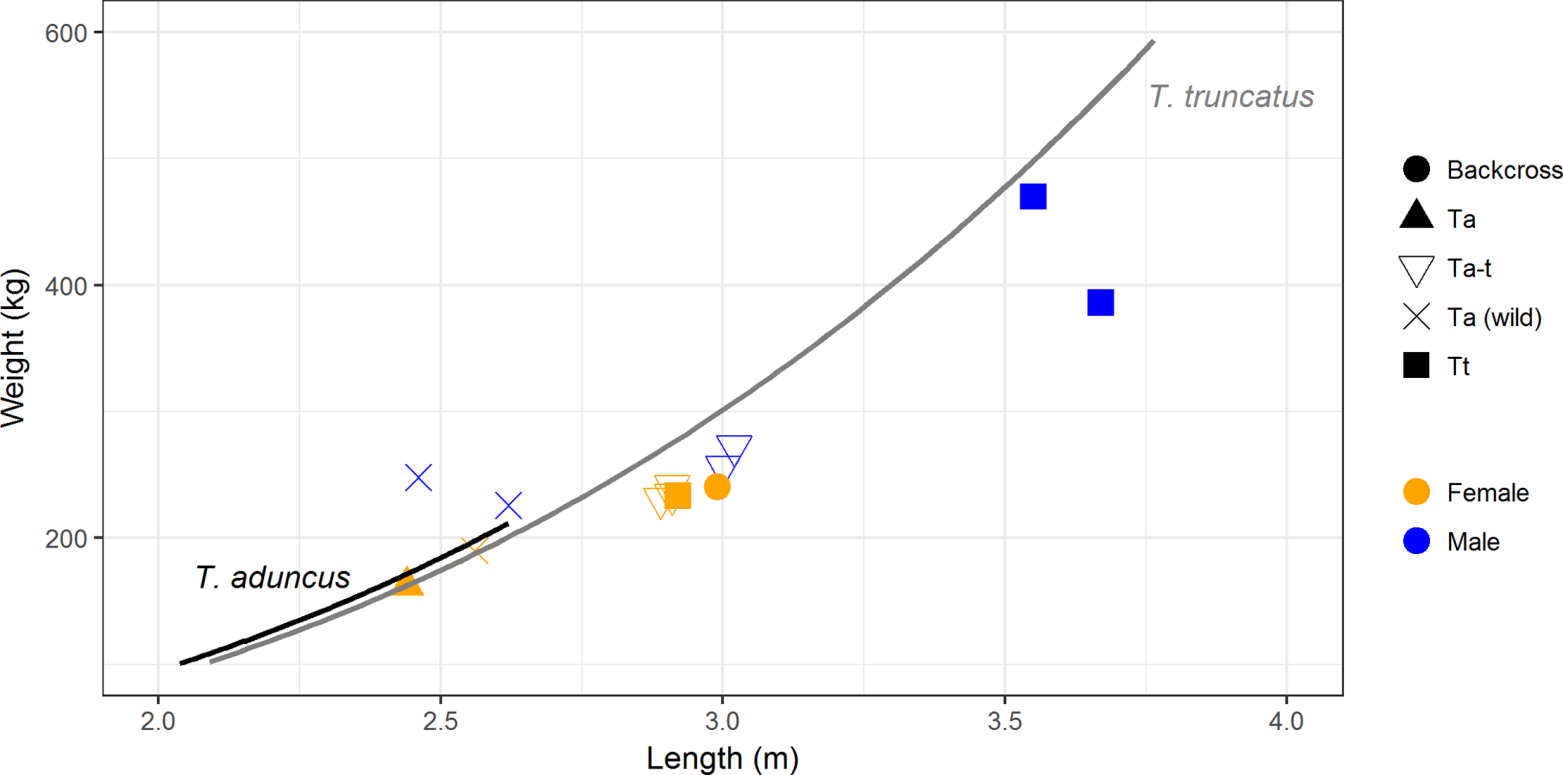
Body length-weight relationship for dolphins housed at uShaka Sea World, as well as examples from the parent *T*. *aduncus* population. Data from three *T*. *aduncus* from KwaZulu Natal are by-caught specimens and the largest examples in the data-set from this region [44]. Growth curves for each species calculated by Best (2007) from 16 common bottlenose (Weight = 11.32 x Length^2.9869^) and 41 Indo-Pacific bottlenose dolphin (Weight = 12.365 x Length^2.9495^) necropsies of animals within the study area.

The length-weight relationships of the hybrid and backcross offspring fall between the parent species (Fig 4). The first generation hybrid offspring (*i*.*e*. all *Ta-t*) have a length of 2.89 to 3.02 m. (mean 2.95 m) and weigh between 231 to 273 kg (mean 247 kg), with BC_1_ falling within this range (2.99 m and 241 kg). The two pure bred male *T*. *truncatus* held at uShaka Sea World are considerably larger (for instance *Tt*_1_ is 3.55 m in length and weighs 470 kgs). However, the pure bred female *T*. *truncatus (Tt*_5_) is unusual in this sample, by having a comparatively small length and weight for the species, attributed to premature maternal separation and restricted development (authors observations). All hybrids are longer and weigh more than *Ta*_1_ and the largest *T*. *aduncus* specimens measured from the wild parent population where *Ta*_1_ originates (Fig 4.).

**Fig 5:**
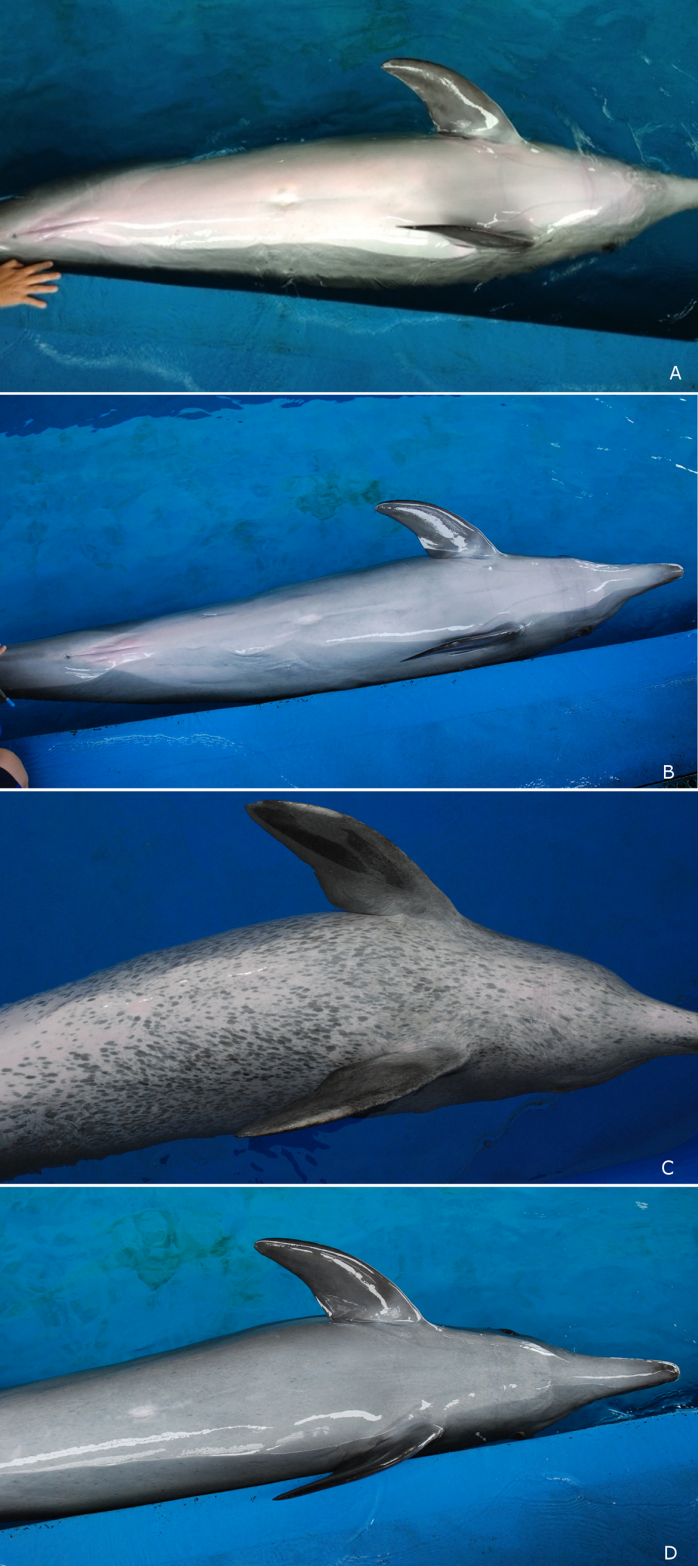
Ventral speckling is a sign of physical maturation in some populations of *T*. *aduncus*. Comparisons of the ventral surfaces of *Ta-t*_7,_ *Ta*_1_ and *Ta-t*_3_ demonstrating degree of ventral speckling or lack thereof. A) *Ta-t*_7_ aged 4 yrs *i*.*e*. before conception, (photo credit S. Pillay), B) *Ta-t*_7_ aged 7 yrs (*i*.*e*. following conception), C) The ventral surface of *Ta*_1_ the *T*. *aduncus* dam of *Ta-t*_7_ at age 42 yrs, D). The oldest *Ta-t* female hybrid at uShaka Sea World displays low levels of ventral speckling at age 23 yrs.

Some *T*. *aduncus* populations exhibit ventral speckling [43, 45], the degree of which increases with age and may indicate sexual maturation. We inspected the ventral surfaces of all dolphins within uShaka Sea World to determine the degree of ventral speckling. Ventral speckling was absent in *Ta-t*_7_ before conception and in 2016 (at age 7 yrs 11 mo) *Ta-t*_7_ still did not exhibit significant ventral speckling (Fig 5A and 5B). In 2016, some ventral speckling was present on the older hybrids held at uShaka Sea World (Fig 5D), although visual assessment indicated a much lesser degree of speckling than considered normal for mature individuals from the parent *T*. *aduncus* or Shark Bay *Tursiops* spp. species [6, 43] (compare Fig 5C and 5D). On the adult hybrids held at uShaka Sea World, the ventral speckles are faint and coverage of the ventral area is sparse (Fig 5D).

Observation and training with the F_1_ hybrids and the surviving backcross hybrid (BC_1_) is ongoing at uShaka Sea World. In all cases, the hybrids are fully incorporated into the daily activities of the facility and demonstrate social and cognitive functions, such as response rates during training for veterinary procedures and strong social bonding, similar to the thoroughbred dolphins housed at the same facility.

## Discussion

To date, most hybridization events in wild cetaceans have been identified through morphological descriptions (e.g. [46, 47]) with the recent application of molecular techniques (e.g. [17, 28, 30, 48, 49]) used to identify hybrids and their parent species. Reports from captive facilities enable the tracking of breeding history (e.g. [50]), and as in our case, can provide important information on the breeding capabilities of dolphin species. Of the odontocetes, the common bottlenose dolphin is the species recorded most frequently to hybridize in captivity [3]. Although there are exceptions [33], the majority of hybrid offspring born in captivity do not survive [3, 21]. Here we demonstrate that F_1_ *T*. *aduncus* and *T*. *truncatus* can survive to adulthood, are healthy and can produce healthy backcross hybrid offspring in cases where the dam is the F_1_ hybrid and the sire is *T*. *truncatus*.

The longevity of the hybrid offspring and most notably the BC_1_ hybrid at uShaka Sea World is unusual amongst captive facilities [3, 21]. This may be explained by the closer taxonomic relationship between *Tursiops* species compared to species involved in inter-generic hybridization events, perhaps facilitating genetic compatibility. Breeding success may also be a reflection of good animal husbandry at the uShaka Sea World captive facility. The apparently normal ovulatory behaviour of the surviving backcross hybrid adult, suggests that subsequent generational hybrids may also be reproductively viable, though the lack of a test for F2 compatibility of hybrids is a limitation, especially since it is often the heterogametic sex (males) that shows hybrid sterility ('Haldane's rule' [51]).

Although rare, there are documented cases of inter-generic hybridization involving *T*. *truncatus*, resulting in fertile hybrids which have subsequently backcrossed with the parent *T*. *truncatus* species. For example, Duffield [52] report that an F_1_ *T*. *truncatus* x *P*. *crassidens* hybrid backcrossed with *T*. *truncatus* on two occasions. In another example, an F_1_ *T*. *truncatus* x *D*. *capensis* hybrid backcrossed with *T*. *truncatus*, however the calf died shortly after birth [33]. Both examples demonstrate the capability for *T*. *truncatus* to hybridize and for the hybrids to backcross. Here we describe in some detail multiple intra-generic hybridization events between *T*. *truncatus* and *T*. *aduncus* and a successful backcross, supporting the potential for this type of reticulation in this genus and the consequent influence on evolution in the wild. We document backcross mating by two parental configurations, and so too few to draw any strong conclusions. We can note however that the parents were unrelated for the offspring that survived (*Tt*_3_ with *Ta-t*_1_, see Fig 1), while the offspring from the inbred mating (*Tt*_1_ with his daughter *Ta-t*_7_) did not.

Data on age at sexual maturity in female *T*. *aduncus* are sparse. Sexual maturity occurs before physical maturity, and earlier in females than males [6, 53]. Timing of maturity may also differ between captive and wild born animals [54] and between geographically separated populations [55], further complicating assessments of reproductive age. For example, mean ovulation age in captive killer whales (*Orcinus orca*) is 7.5 years and age at first conception 9.8 years, compared to the average first conception age of 12.1 years in wild, free ranging populations [56]. In the wild, ovulation in female *T*. *aduncus* from South African waters is reported to take place between 9.5 and 11 years of age [6]. However, reports of a stranded female from an earlier study suggest that sexual maturity can be attained under 9 yrs of age, and possibly as early as 6 yrs [42]. There are reports of sexual maturity as early as 3.5 years in *Tursiops* from Japan [57]. However, these data are derived from the examination of deceased dolphins, and it is unclear whether this minimum age is based on the occurrence of *corpora lutea* in the ovaries or observed pregnancies in animals of this young age (or both), with no further data on whether the outcome of pregnancy was a viable offspring [57]. Data from free ranging *T*. *truncatus* from Namibia are similarly sparse, although there is evidence from this population that first conception can take place around 5.5 years of age [58] and at approximately 2.8 m total length [38]. There are few data on the age at maturity of hybrids and whether, like other morphological [22, 25, 33, 38] and behavioural [25] characteristics, it is intermediate between that of the parent species. Zornetzer and Duffield [33], for example report the birth of a calf to a hybrid *T*.*truncatus* x *D*.*capensis*, born when the dam was 7.5 yrs and presumably conceived around 6.5 yrs of age. Our data on pregnancy in F_1_ *T*. *aduncus* x *T*. *truncatus* hybrids demonstrates that these animals can become pregnant early in life compared to the parent species. The estimated age of conception of 4 years and 3 months reported here for *Ta-t*_7_ may therefore be the youngest known viable pregnancy for either parent *Tursiops* species or hybrid thereof.

That *Ta-t*_7_ was still observed nursing during the period of conception is also of interest. Bottlenose dolphins can begin ingesting solid food between 4 and 11 months of age [59], with a combined solid and milk diet thereafter. At uShaka Sea World, *Ta-t*_7_ began eating solids from 4.5 months onwards. Bottlenose dolphins and other odontocetes are known to have prolonged lactation [59] and in South African *T*. *aduncus* milk remains have been documented in the stomachs of calves up to three years of age [60]. Although the majority of calves from bottlenose dolphins from Shark Bay, Western Australia were weaned before four years, some continued to suckle after this, with one animal only weaned at eight years of age [61]. Lactation in mammals, including dolphins, relies on close proximity and physical stimulation of the mammary area [62–64]. Captive studies have demonstrated that persistent suckling attempts can induce lactation when orphaned calves are held in close proximity to previously non-lactating *Tursiops* females [65]. In the wild, pre-weaned animals maintain a close association with their mother, with weaning initiated during the females' next pregnancy [61]. Therefore, the close association of mother and calf in the captive facility may have prolonged the lactation period of *Ta*_1_ to four years of age and beyond.

Morphological characteristics of hybrid cetacean offspring appear intermediate to the parent species [3, 33]. In the wild *T*. *aduncus* are smaller in length and estimated weight compared to *T*. *truncatus* [38]. Although limited, our length-weight data indicate that the size of hybrid offspring is intermediate to the biological parents, indicating it falls intermediate between the parent species (Fig 3). This observation might help identification of hybrids in the wild, however a greater sample size including unrelated individuals would clarify this relationship. The coloration patterns of hybrids can also differ from parent species, usually being somewhat intermediate [22, 33, 38]. Ventral speckling is absent in *T*. *truncatus* but is prominent in some populations of *T*. *aduncus* and the *Tursiops* spp. population found in Shark Bay, Western Australia which have had an uncertain taxonomic status but speckling patterns similar to *T*. *aduncus* [6, 43]. In the latter population, speckling develops with age, first appearing around the genital area around 10 years of age, but can occur as early as 7 years. The age of speckle onset around the genitalia usually correlates with the age of first parturition and is considered an honest sign of sexual maturation in the Shark Bay population [43]. The development of speckling has not yet been determined in hybrid *Tursiops* dolphins. Our observations indicate that the onset or degree of ventral speckling is not a reliable indicator of sexual maturity in F_1_ *Tursiops* hybrids.

Karyological similarity within the Cetacea (most have the same number of chromosomes: 2n = 44 [3]) has been proposed as one explanation for the apparent ease with which distinctly related cetacean species hybridize [66]. Where their distributions overlap, new cetacean species can originate through hybridization, as demonstrated for the Clymene's dolphin [4] and environmental pressures such as climate change may increase the frequency of introgressive hybridization, as recently suggested for pilot whales, genus *Globicephala* [32]. The distribution of *T*. *aduncus* and *T*. *truncatus* occur in parapatry throughout the Indo-Pacific region, with sympatric distributions in some areas such as the waters off South East China [8]. Given that we have demonstrated several hybridization events, it is somewhat surprising that other hybridization events have not been documented in wild populations and the genetic integrity of the parent species remains intact in areas where their distributions overlap such as in the Taiwan Strait [8, 67] and Australia [16]. Indeed, relatively high levels of genetic isolation have been documented in such areas [67]. Behavioural isolation mechanisms may be operating in the wild to reduce hybridization events. For example, *T*. *aduncus* and *T*. *truncatus* produce acoustic communication signals (whistles) with distinguishable frequency compositions [68, 69], which could assist in inter-species recognition thereby reducing intra-generic mating attempts.

## Conclusion

We have demonstrated that *T*. *aduncus* x *T*. *truncatus* F_1_ hybrids can survive to adulthood, are healthy and can produce healthy backcross hybrid offspring. The documented hybridization in captivity may be an artefact of the close proximity and the limited mating opportunities afforded by captive situations, limiting mate choice and assortative mating. However, low levels of intra-generic hybridization in *Tursiops* may well be taking place in the wild [34], and may be revealed following more extensive molecular screening in the relevant geographic regions.
